# Prevalence and phylogenetic analysis of hepatitis E virus in pigs in Vietnam

**DOI:** 10.1186/s12917-020-02537-7

**Published:** 2020-09-14

**Authors:** Hu Suk Lee, Duy Tung Dao, Vuong Nghia Bui, Ngoc Anh Bui, Thanh Duy Le, Hung Nguyen-Viet, Delia Grace, Krishna K. Thakur, Katsuro Hagiwara

**Affiliations:** 1 International Livestock Research Institute (ILRI), Regional Office for East and Southeast Asia, Room 301-302, B1 Building, Van Phuc Diplomatic Compound, 298 Kim Ma Street, Ba Dinh District, Hanoi, Vietnam; 2 National Institute of Veterinary Research, Hanoi, Vietnam; 3 International Livestock Research Institute (ILRI), Nairobi, Kenya; 4 Natural Resources Institute, Chatham, UK; 5 Department of Health Management, Atlantic Veterinary College, University of Prince Edward Island, Charlottetown, Canada; 6 School of Veterinary Medicine, Rakuno Gakuen University, 582 Bunkyodai, Ebetsu, Hokkaido 069-8501 Japan

**Keywords:** Vietnam, pigs, Hepatitis E, prevalence, phylogenetic analysis

## Abstract

**Background:**

Hepatitis E virus (HEV) is a zoonotic disease and has been reported around the world. The main objective of this study was to evaluate the sero-prevalence and phylogenetic analysis of HEV in Vietnam. Pig blood and fecal pooled samples were collected to assess the prevalence of HEV. We assessed the true prevalence (TP) of HEV from apparent prevalence (AP) by taking into account the sensitivity and specificity of diagnostic tests using a Bayesian approach. For phylogenetic analysis, the data compared with worldwide HEV reference strains including all eight genotypes (G1-G8) which were identified in previous study.

**Results:**

A total of 475 sera and 250 fecal pooled samples were collected at slaughterhouses and pig farms from five provinces, in Viet Nam. Overall, the sero-AP of HEV was 58.53% (95% confidence interval: 53.95–62.70) while the sero-TP was slightly higher (65.43, 95% credible interval: 47.19–84.70). In terms of pooled samples, overall, the RNA-AP was 6.80% (95% confidence interval: 4.01–10.66). One strain in Hanoi, two strains in Dak Lak, seven strains in An Giang, four strains in Son La and two strains in Nghe An were isolated. The phylogenetic tree demonstrated that 19 Vietnamese strains were clustered into HEV 3 and 4.

**Conclusions:**

This study provided evidence that HEV is circulating in domestic pigs in Vietnam. From a public health perspective, it is very important to raise public awareness for high-risk groups (e.g. slaughterhouse workers, pig traders, farmers and market sellers) who have more opportunities to come in contact with pig and contaminated meats.

## Background

Hepatitis E virus (HEV) is one of the important zoonotic diseases with a worldwide distribution, and it is commonly reported in Asia, Africa and Latin America [1]. Annually, 20 million HEV infections are reported around the world, with cases in South and Southeast Asia accounting for 60.6% or the total and deaths 64.7% [1, 2]. The virus has been classified into the *Orthohepevirus* genus within the *Hepeviridae* family [3]. Currently, at least eight genotypes of HEV have been identified [4, 5]. Five genotypes (HEV 1–4 and 7) are transmitted from human to human primarily via the fecal-oral route due to fecal contamination of drinking water [1, 6]. In general, these genotypes are commonly circulating in the areas with poor sanitation and low socio-economic status. Genotypes 3 and 4 have been considered a foodborne zoonotic disease that is commonly transmitted to humans by ingestion of raw or undercooked meat products (e.g. pig, wild boar and deer), drinking of animal milk (e.g. camels) and via direct exposure to animal feces [6–8].

Previous studies have isolated HEV 3 and 4 from pigs in the United States, the United Kingdom, France, South Korea, China and Japan [9–14]. Phylogenetic study provided evidence that the swine-origin HEV and human HEV isolates had a close association, suggesting that pigs play an important role in the transmission of the virus to humans [15].

In Southeast Asia, some studies revealed the transmission of HEV 3 and 4 from animals to humans providing evidence of circulating HEV in pigs [16–18].

In Vietnam, the first HEV outbreak in humans was reported in 1994 along the river bordering Cambodia in the southwestern part [19]. One study showed that HEV is circulating in pigs (prevalence: 19.1% in fecal samples and 8.2% in rectal samples) while humans showed 31.7% seroprevalence [20]. In addition, a recent study in Vietnam found that pig farmers, slaughterhouse workers and pork meat retailers had high seroprevalence compared to unexposed groups [21]. Also, it showed that HEV was detected from liver tissues in pigs.

To our knowledge, no studies have been conducted to evaluate the true prevalence (TP) of HEV in pigs in Vietnam. TP is the proportion of animals which have a disease in the population while apparent prevalence (AP) is the proportion of animals testing positive by a diagnostic test. If sensitivity and specificity of diagnostic test are less than 100%, estimated prevalence is biased. We therefore used a Bayesian analysis to estimate the TP from AP. The main objective of this study was to assess the TP and phylogenetic analysis of HEV in pigs from 5 provinces in Vietnam.

## Results

### Estimated true prevalence of HEV

A total of 475 sera samples were collected at slaughterhouses from five provinces. Overall, the sero-AP of HEV was 58.53% (95% CI: 53.95–62.70) while the sero-TP was slightly higher (65.43, 95% credible interval: 47.19–84.70) (Table 1). Dak Lak had the highest sero-prevalence followed by Nghe An and Son La whereas An Giang and Hanoi showed the lowest sero-prevalence. Overall, the sero-TPs were higher than the sero-APs except for An Giang and Hanoi provinces. Brooks-Gelman-Rubin (BGR) plots displayed that all chains converged for sero-TPs (S1 Fig).

**Table 1:** Sero-apparent prevalence with 95% CI ^a^ and sero-true prevalence with 95% credible interval for hepatitis E virus in sera samples in pig slaughterhouses

Province (no.)	Positive samples	AP (%) with 95% CI^a^	TP (%)^b^ with 95% credible interval
An Giang (95)	20	21.05 (13.36–30.62)	8.04 (0.02–29.56)
Dak Lak (95)	86	90.53 (82.78–95.58)	97.24 (86.39–99.99)
Hanoi (95)	26	27.37 (18.72–37.48)	15.21 (0.09–38.66)
Nghe An (95)	75	78.95 (69.38–86.64)	93.19 (72.39–99.99)
Son La (95)	71	74.74 (64.78–83.09)	90.57 (67.09–99.98)
Total (475)	278	58.53 (53.95–62.70)	65.43 (47.19–84.70)

^a^ *CI* confidence interval; ^b^ Median value was recorded

A total of 250 pooled fecal samples were collected at farm level. Overall, the RNA-A was estimated at 6.8% (95% CI: 4.01–10.66%) (Table 2). An Giang had the highest RNA-AP followed by Son La while Hanoi showed the lowest RNA-AP. There were no statistically significant differences in sero-prevalence between female (79.10, 95% CI: 73.74–83.81) and male (79.23, 95% CI: 73.06–84.54) pigs as the confidence intervals of the groups were overlapping.

**Table 2:** RNA-apparent prevalence with 95% CI ^a^ for hepatitis E virus in fecal pooled samples in pig farms (2 fecal pooled samples / farm)

Province (no.)	Positive pooled samples	RNA-apparent prevalence with 95% CI ^a^
An Giang (50)	6	12.00 (4.53–24.31)
Dak Lak (50)	3	6.00 (1.25–16.55)
Hanoi (50)	1	2.00 (0.05–10.65)
Nghe An (50)	3	6.00 (1.25–16.55)
Son La (50)	4	8.00 (2.22–19.23)
Total (250)	17	6.80 (4.01–10.66)

^a^ *CI* confidence interval

### Phylogenetics analysis of HEV

**Fig. 1:**
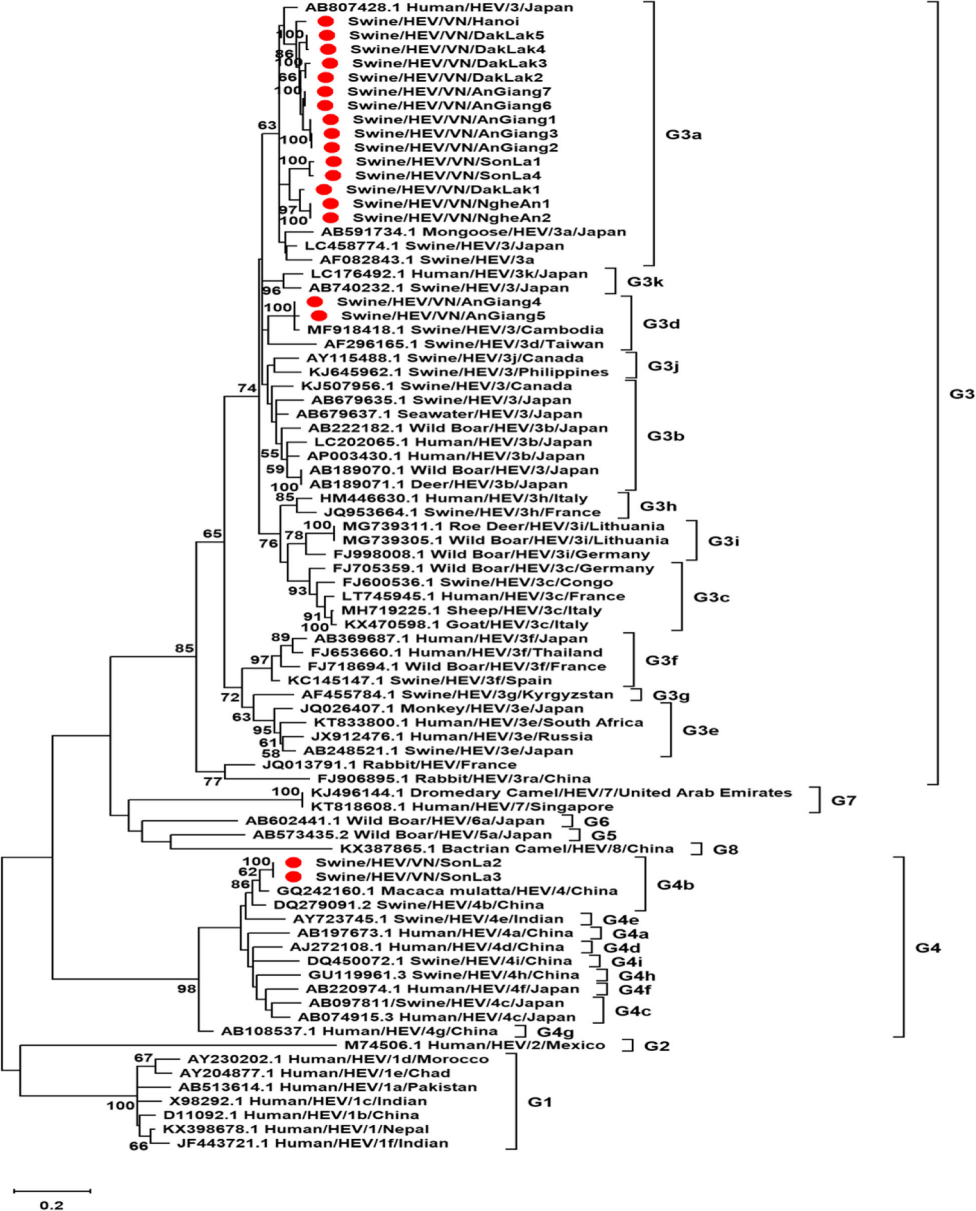
Maximum-likelihood tree constructed for 348 bp HEV ORF2 nucleotide sequences (nt position 6022–6369) of 19 Vietnamese strains (indicated by red dot ), and reference strains obtained from the GenBank database. Genotype G1-G8 are indicated on the right of the figure. The bar at the bottom of the figure denotes evolutionary distance

The 348 bp HEV ORF2 nucleotide sequence was successfully obtained from fecal samples. A total of 19 Vietnamese strains were isolated in samples, including 1 strain in Hanoi, 5 strains in Dak Lak, 7 strains in An Giang, 4 strains in Son La and 2 strains in Nghe An (Fig. 1). All 19 nucleotide sequences of HEV ORF2 have been deposited on GenBank (accession number MT670024–MT670042). The phylogenetic tree demonstrated that 19 Vietnamese strains were clustered into HEV 3 and 4 among 8 genotypes. Genotype 3 is dominant including 17 strains divided into two sub-genotypes 3 (15 strains (MT670024-MT670034, MT670037-MT670039, MT670042) belonged to sub-genotype 3a and 2 strains (MT670035, MT67036) clustered into sub-genotype 3d). Two remaining strains (MT670040, MT670041) were identified from Son La province were clustered into HEV 4, sub-genotype 4b. These strains showed a close relationship with the strain isolated from a monkey in China.

**Fig. 2:**
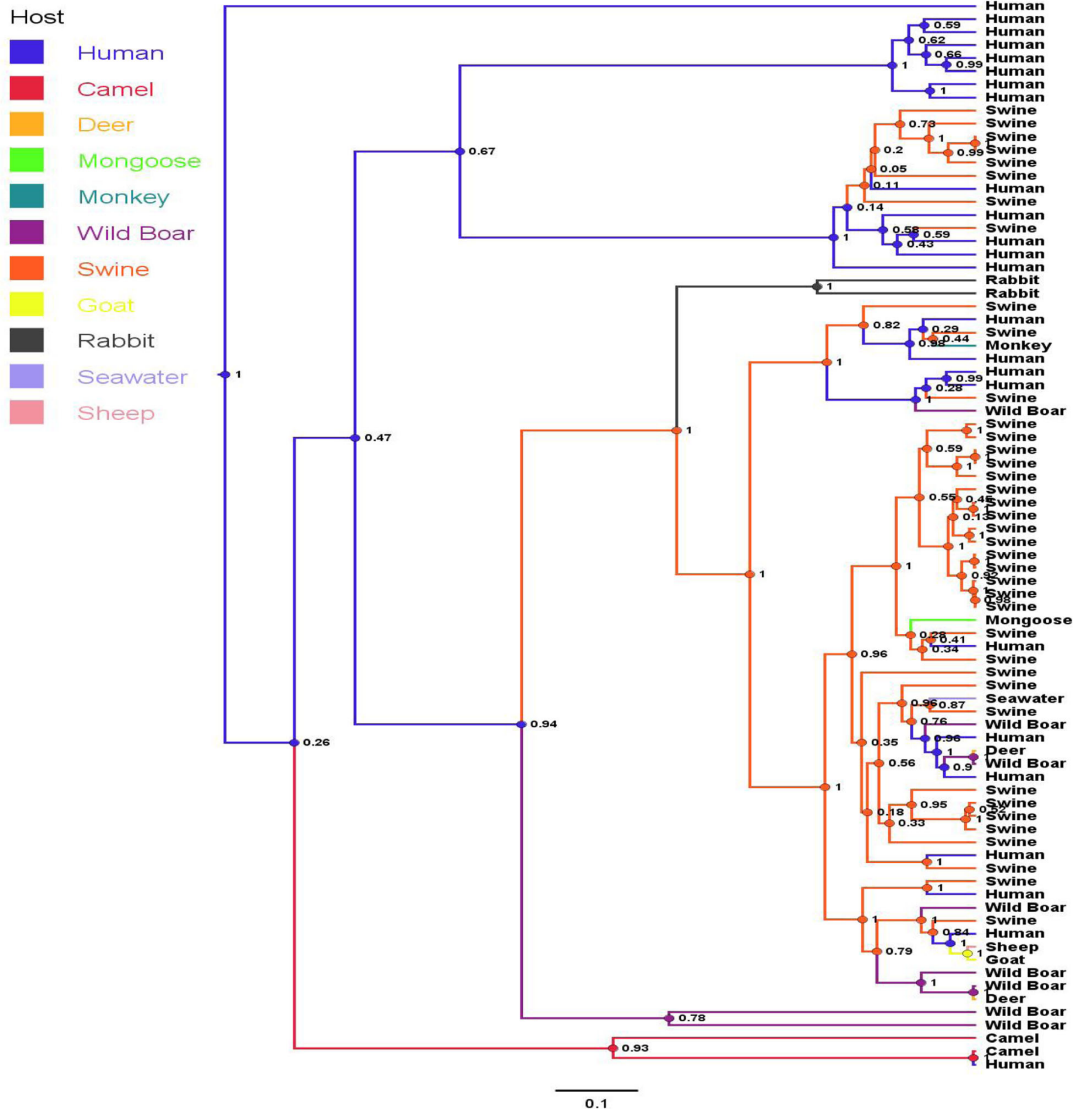
The Bayesian maximum clade credibility (MCC) host discrete traits tree for 348 bp HEV ORF2 nucleotide sequences (nt position 6022–6369) of 19 Vietnamese strains, and reference strains obtained from the GenBank database. The phylogenetic host tree indicated the transmission between HEV hosts, the host at node indicated the ancestor of sub-group and the number at the node indicated the posterior probability. The bar at the bottom of the figure denotes evolutionary distance

Based on the host phylogenetic tree, it is assumed that HEVs are human in origin (Fig. 2). Humans are the main reservoir that spread the virus to other animals including swine, wild boar, goat, and camel. However, humans can also be infected with HEVs from swine and camel. Swine play an important role in transmitting the HEVs to mongoose, monkey, wild boar and in contaminating the environment (seawater). In addition, the host phylogenetic tree indicated the HEVs can be transmitted from goats to sheep and from wild boar to deer.

## Discussion

A large national study was implemented to evaluate the prevalence and molecular characteristics of HEV in pigs across Viet Nam. Overall, our anti-HEV IgG prevalence (approximately 58%) was very similar to other studies in Germany (47%), Italy (50.2%), Japan (56%), Philippines (50.3%) and Laos (51.2%) [22–26]. Some studies showed a higher seroprevalence of IgG as pigs grow older [27, 28]. However, we were not able to evaluate this because sera samples were obtained from fattening pigs (6–8 months old and weighing 60–120 kg) at slaughterhouses. Interestingly, sero-TPs of three provinces (Dak Lak, Nghe An and Son La) was estimated more than 90%, suggesting a high potential zoonotic transmission to humans. Overall, the RNA-positive rate (6.8%) in pooled fecal samples was very similar to other studies in Laos (11.5%), Philippines (7.4%) and Thailand (1.27–2.9%) [18, 22, 29, 30].

Previous studies have provided evidence that there was an epidemiological association between consumption of pork meat and HEV cases [31, 32]. In Vietnam, most of the farmers in rural areas raise pigs in backyards with poor sanitary conditions. They have more opportunities to come in contact with pigs and pig waste. In addition, it is very common that local people consume uncooked pork, livers meats, sausages, unwashed vegetables and insufficiently treated drinking water. Therefore, it might be possible that people who are exposed to pig and pork meats are at an increased risk of HEV transmission [33]. Further studies are needed to evaluate whether this exposure route poses a public health concern as well as to better understand the human behavior for preventing HEV infections.

The Bayesian maximum clade credibility (MCC) host discrete traits tree provided information about the transmission of HEVs between humans, swine and other animals. Humans are the main reservoir/ maintenance host of HEV but pigs are a well-known as a reservoir of HEV that transmit the virus to humans. In addition, the host phylogenetic tree indicated that HEV can be transmitted from camel to humans [8]. This tree showed that HEV genotypes 3 and 4 are considered zoonotic viruses, and the new HEV genotype 7 can be transmitted from camel to humans. Therefore, HEVs are now recognized as an emerging zoonotic agent.

We found that both genotypes HEV 3 and 4 were detected in domestic pigs across the country which was in line with other Asian countries [34–36]. Most of them (15/19) were classified into the existing genotype 3 which is the commonly detected subtype in Asia (including Japan, South Korea and Philippines) [22, 37, 38]. The two remaining Vietnamese HEV strains that were isolated in An Giang, were clustered into sub-genotype 3d with Cambodia and Taiwan strains. The HEV 4 (sub-genotype 4b) was only detected in Son La province which is on the borders with Laos [18, 34, 39]. Therefore, it might provide evidence that this type is circulating in these areas. The proportion of minorities (e.g. Thai and H’Mong) is relatively higher than areas in Son La province (the major ethnic group is Kinh, occupying 85.7% of the population) [40]. In general, ethnic minorities are less educated and incomes than the Kinh group, which are associated with limited awareness of hygiene and food safety in Vietnam [41]. In addition, other studies found that highly educated people had a better knowledge of food safety as opposed to those with low education [42, 43]. Ethnic minorities may benefit from efforts to enhance public awareness of food safety and disease prevention. Further epidemiological study needs to be conducted to establish a transmission link between pigs and humans in Son La province. Also, one recent study showed that the HEV seroprevalence was higher among individuals occupationally exposed to pig and pork products [21]. Therefore, the human health impact of HEV should be properly defined to establish appropriate interventions.

The main limitation of our study was that our blood and fecal samples may not be representative because blood samples were only collected at slaughterhouses and were disproportionally collected depending on the number of pigs at each farm. Large and medium scale farmers were not willing to participate (mainly for biosecurity reasons) in our study. Although, our pig samples were not representative of the general population, we think that our findings provide valuable information on the epidemiology of HEV in Vietnam. It could be possible that the TP of HEV may be under/overestimated due to a random error with our measuring facilities. In addition, our prior estimates for the sensitivity and specificity of the ELISA test were obtained from our experiment and therefore, we are not clear which estimates are suitable for the Vietnamese context. We conducted some simulations to explore what happens with *N* = 80, 70, 60, etc. out of 95 samples. There was a large discrepancy between the AP and TP as the positive cases decreased. The main reason was that when data (likelihood) is weak, posterior estimates are strongly influenced by priors [44].

A Bayesian method provides a chance to integrate prior information with observed data, which is useful for estimating values for both prevalence and diagnostic characteristics of tests [45]. If a diagnostic test has less than 100% sensitivity and specificity, the estimated prevalence is likely to be biased. Therefore, a Bayesian approach was applied to estimate the TP from AP. Overall, Bayesian models are very susceptible replying on the priors, so we employed a Jeffreys prior to reduce the impact of prior to the posterior distribution as there was no prior information for HEV prevalence in the study sites. This study was the first attempt to evaluate the TP for HEV in Vietnam and demonstrates how a Bayesian analysis can be applied to better estimate the prevalence of diseases.

## Conclusion

This study provided evidence that HEV is circulating in domestic pigs in Vietnam. Further study is necessary to evaluate the possible transmission from pigs and environmental risk factors to humans. From a public health perspective, it is very important to raise public awareness for high-risk groups (e.g. slaughterhouse workers, pig traders, farmers and market sellers) who may have more opportunities to come in contact with pig and contaminated meats. Therefore, a national surveillance system and practical guidelines for proper handling of meat products needs to be established for disease control and prevention.

## Methods

### Study locations and sampling

**Fig. 3:**
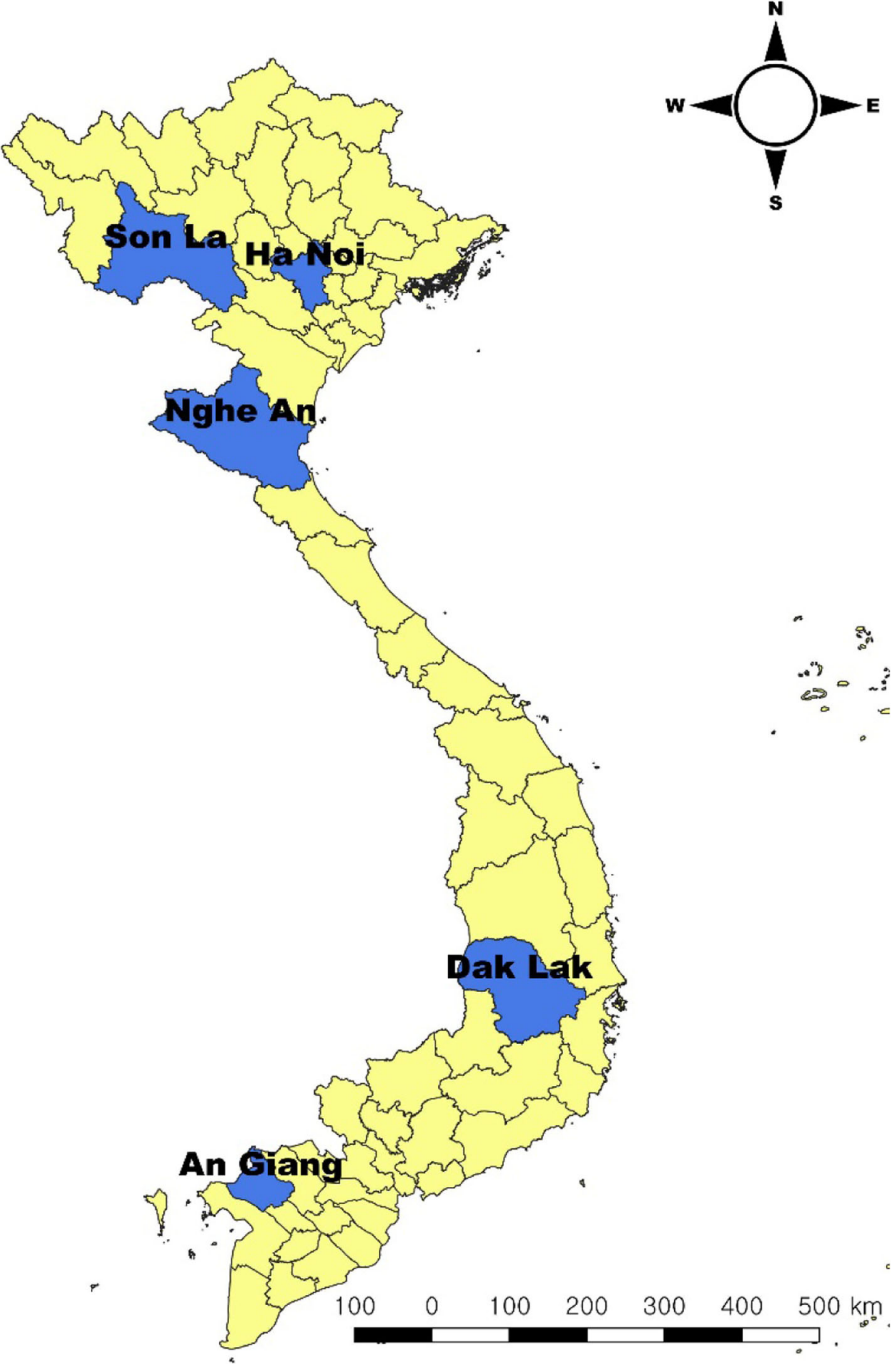
Five sampling provinces for HEV in pigs in slaughterhouses and farms. *This map was created by our own

Pig blood samples from previous cross-*sectional* survey [46] were used to estimate the sero-prevalence of HEV at the National Institute of Veterinary Research (NIVR) in Hanoi. Sera samples from fattening pigs (6–8 months old and weighing 60–120 kg) were randomly collected from the jugular vein at slaughterhouses in five provinces (An Giang, Dak Lak, Hanoi, Nghe An, Son La) (Fig. 3). For our study, 95 sera samples were randomly selected from each province (385 samples / province). Fecal pooled samples were collected in 25 farms/province from 2018 to 2019. Two fecal pooled samples of a minimum of 2 g were collected in a sterile plastic container at each farm and were kept at − 20 °C until transportation. This study was approved by the Hanoi Medical University Institutional Review Board (IRB: no. 00003121), Vietnam.

### Laboratory analysis

#### Serum examination

Anti-HEV antibodies were detected by enzyme-linked immunosorbent assay (ELISA) using HEV genotype 3 -derived VLP as an antigen. For the detection of antigen bound IgG, anti-pig IgG antibody-HRP (Bethyl Laboratories Inc. TX. USA) conjugate was applied as a secondary antibody. The serum samples were diluted 1:100 with PBS containing 0.05% Tween 20, and 10% of Block Ace (DS Pharma Promo Co. Ltd. Osaka, Japan), and incubated for 1 h at room temperature. After the secondary antibody reactions, 50 μl of TMB (3,3′,5,5′-tetramethylbenzidine) (Kirkegaard & Perry Laboratories Inc., Baltimore, MD, USA) was added, and after a 10-min incubation at room temperature, 50 μl of 2 M sulphuric acid was added to stop the reactions. The optical density (OD) value at 450 nm was measured by a microplate spectrophotometer (Thermo Fisher Scientific Inc. USA). The Index value was calculated from the OD value obtained by ELISA as follows.

$$ \mathrm{Index}\ \mathrm{value}:\frac{\mathrm{Sample}\ \mathrm{OD}-\mathrm{Control}\ \mathrm{OD}}{\mathrm{Positive}\ \mathrm{OD}-\mathrm{Control}\ \mathrm{OD}}\ \mathrm{x}\ 100\ \left(\%\right) $$

The cut off value was set at 0.295 by calculating the average value + 2 standard deviation of negative samples (*n* = 5). As a result, the sensitivity and a specificity for the test were 90.0% (95% CI: 68.30–98.77) and 91.67% (95% CI: 73.0–98.97) based on the test results of 20 experimentally infected and 24 Negative pigs.

#### RNA extraction and detection of HEV by RT-PCR

A portion of fecal samples was suspended with PBS and centrifuged at 5000 rpm for 5 min, and 250 ul of the supernatant was used for RNA extraction. The RNA was extracted using the RNA purification kits via the Direct-zol™ (Zymo Research, CA. USA) method. One-step RT-PCR using HEV ORF2 as a target region was performed using the extracted RNA as a template. The QIAGEN One-Step RT-PCR Kit (QIAGEN) was used for RT-PCR master mix using 5 μl of RNA as a template, and 10 μM each primers Her F1: 5′-AATTATGCYCAGTAYCGRGTTG-3′ and Her R1: 5′-CCCTTRTCYTGCTGMGCATTCTC-3′ [9]. The reaction conditions were reverse transcription at 50 °C for 30 min, and then at 95 °C for 15 min. As a PCR cycle, denaturation was performed at 94 °C for 30 s, annealing at 55 °C for 30 s, and extension at 72 °C for 1 min for 40 cycles. Next, nested-PCR was performed using the product obtained by RT-PCR as a template. A total of 10 μM primers (Her F2:5′-GTWATGCTYTGCATWCATGGCT-3′ and Her R2:5′-AGCCGACGAAATCAATTCTGTC-3′) and TaKaRa ExTaq (Takara Bio Inc., Japan) were used. The reaction conditions were: preheating at 95 °C for 2 min, 35 cycles of denaturation at 94 °C for 30 s, annealing at 55 °C for 30 s, and extension at 72 °C for 30 s as one amplification cycle, followed by extension at 72 °C for 5 min. The product of nested-PCR was electrophoresed on a 1.5% agarose gel, and an amplified band of HEV-RNA was detected. The expected amplified band (348 bp) was sequenced after purification.

#### DNA sequencing

Two PCR products from each DNA sample were sequenced in both directions using the same nested PCR primers (Her F2, Her R2) by ABI Prism Big Dye Terminator v3.1 Cycle Sequencing Kit (Applied Biosciences, Foster City, CA, USA). The sequences were analyzed by 3500 Genetic Analyzer (Life Technologies, Carlsbad, CA, USA). The consensus sequences were generated from two bidirectional repeats of each sample by software GENETYX Ver.13.0.3 (GENETYX Corp., Tokyo, Japan).

### Data analysis

We evaluated the true prevalence (TP) of HEV from apparent prevalence (AP) by taking into account the sensitivity and specificity of diagnostic tests using a Bayesian approach [47]. For sera samples, the prior distribution for sensitivity [95 CI% (0.683, 0.988)] and specificity [95% CI (0.73,0.99)] were estimated from the ELISA experiments. For the TP, a non-informative Jeffreys prior [beta = (0.5, 0.5)] was used to minimize the influence on the posterior [48]. For fecal pooled samples, we were not able to calculate the TP because the sensitivity and specificity of PCR were not reported from the previous research experiments. Markov Chain Monte Carlo (MCMC) sampling was implemented by JAGS through the rjags package using the in “truePrev” function in the package prevalence in R [49, 50]. The first 1000 samples of the three MCMC chains were discarded as a burn-in period and the following 10,000 iterations were used for posterior inference. The AP was calculated with a 95% Clopper-Pearson/Exact confidence interval (CI). The TP was estimated based on the posterior median value with a 95% credible interval. The statistical significance of the differences between prevalence estimates was assessed by examining the overlap of the respective credible intervals. The outputs from the three chains were visually evaluated using MCMC trace-plots, posterior density distribution plots, Brooks-Gelman-Rubin (BGR) plots, and auto-correlation plots using the CODA package [51]. All data were imported into Microsoft Excel 2016 and analyzed using R (version 3.6.2). QGIS (Quantum GIS Development Team 2018. QGIS version number 3.8.3) was used to create the map.

### Phylogenetic analysis

The 348 bp HEV ORF2 nucleotide sequences obtained in this study were aligned using CLUSTALW multiple alignments in BioEdit version 7.2.5. The data compared with worldwide HEV reference strains including all eight genotypes (G1-G8) which were identified in previous study [52]. In addition, phylogenetic analyses were conducted using the Maximum Likelihood (ML) method by MEGA X software [53] with parameter settings of 1000 bootstrap replicates, and the best fit model GTR + G + I.

The same data was utilized to generate ML phylogenetic tree, which was initially used to conduct the Bayesian maximum clade credibility (MCC) host discrete traits tree by using software BEAST v1.8.4 (http://tree.bio.ed.ac.uk/software/beast/). The strict clock and the best fit GTR + G + I nucleotide substitution model with a constant population size coalescent tree prior were used. The MCMC was run at 50,000,000 generations and sampled at every 5000 generations. The effective sample size (ESSs) of the analysis was checked by software Tracer v1.6 (http://tree.bio.ed.ac.uk/software/tracer/). The MCC host discrete traits output tree was generated by using TreeAnnotator v1.10.4 (http://tree.bio.ed.ac.uk/software/beast/) afterburn 10% of the first trees. The host phylogenetic tree was reconstructed by software FigTree v.1.4.3 (http://tree.bio.ed.ac.uk/).

## Supplementary information

**Additional file 1.**

## Data Availability

The datasets generated and/or analyzed during the current study are available in the GenBank repository, Accession number (MT670024–MT670042) to datasets.

## Abbreviations

HEV: Hepatitis E virus
NIVR: National institute of veterinary research
ELISA: Enzyme-linked immunosorbent assay
OD: Optical density
RT-PCR: Real time – polymerase chain reaction
TP: true prevalence
AP: apparent prevalence
MCMC: Markov Chain Monte Carlo
BGR: Brooks-Gelman-Rubin
MCC: maximum clade credibility
ESS: effective sample size
