# Supplementary material for: Prevalence and phylogenetic analysis of hepatitis E virus in pigs in Vietnam
Source: BMC Vet Res. 2020 Sep 14;16:333. doi: 10.1186/s12917-020-02537-7 (PMC7489210; doi:10.1186/s12917-020-02537-7)

Supplementary 1.

The BGR plots displayed that all chains have converged for TPs – pooled samples

An Giang


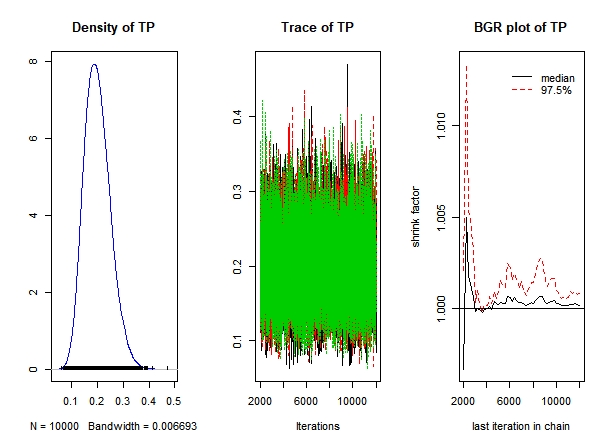


Dak Lak


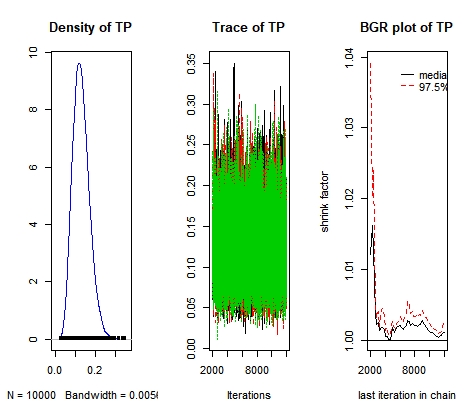


Hanoi


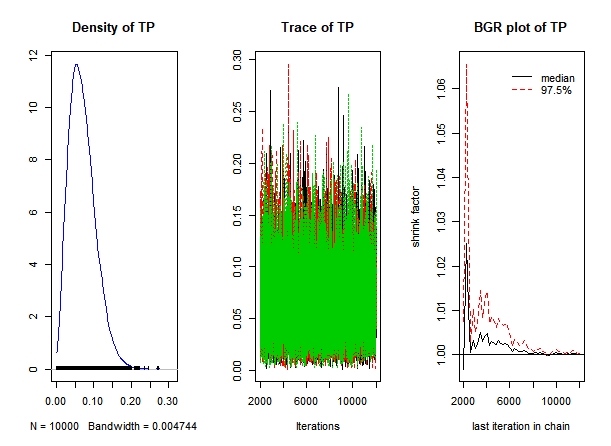


Nghe An


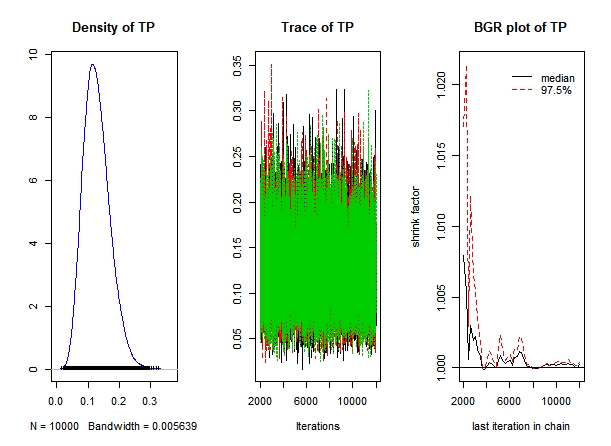


Son La


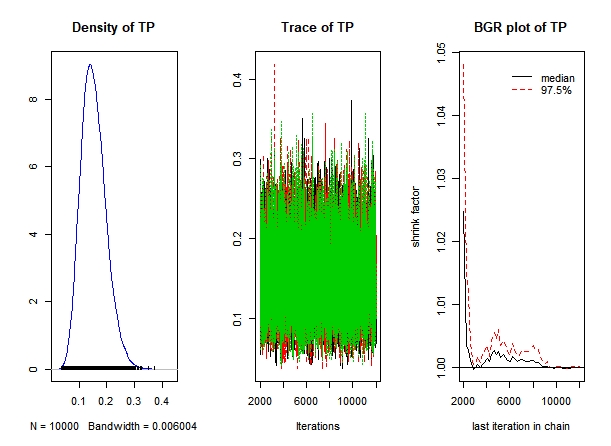


The BGR plots displayed that all chains have converged for TPs – sera samples

An Giang


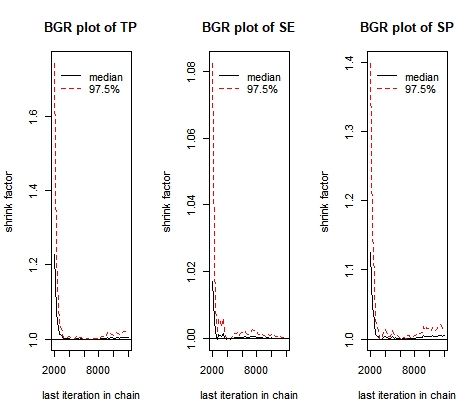


Dak Lak


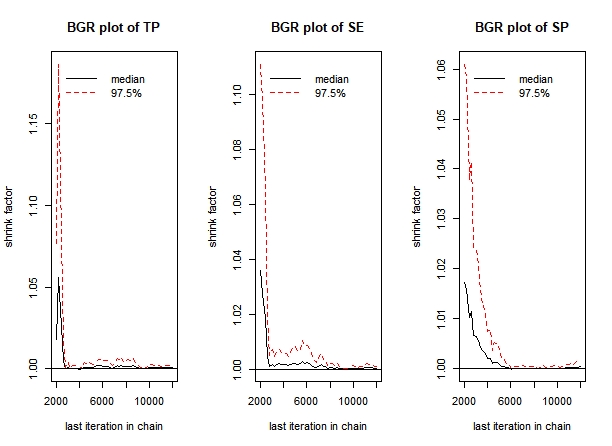


Hanoi


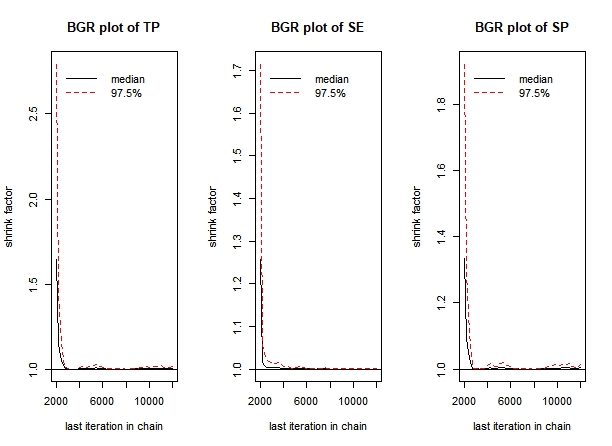


Nghe An


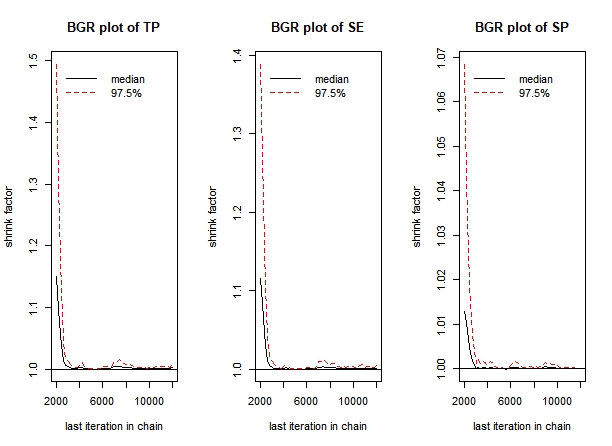


Son La


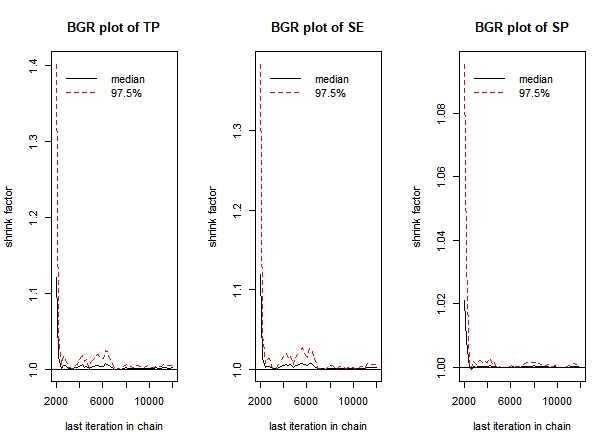

Supplement: Supplementary file 1 — Additional file 1. [file 12917_2020_2537_MOESM1_ESM.docx]
